# (Why) Do You Like Scary Movies? A Review of the Empirical Research on Psychological Responses to Horror Films

**DOI:** 10.3389/fpsyg.2019.02298

**Published:** 2019-10-18

**Authors:** G. Neil Martin

**Affiliations:** Faculty of Humanities, Arts and Social Sciences, School of Psychotherapy and Psychology, Regent’s University London, London, United Kingdom

**Keywords:** horror, terror, fear, film, cinema

## Abstract

Why do we watch and like horror films? Despite a century of horror film making and entertainment, little research has examined the human motivation to watch fictional horror and how horror film influences individuals’ behavioral, cognitive, and emotional responses. This review provides the first synthesis of the empirical literature on the psychology of horror film using multi-disciplinary research from psychology, psychotherapy, communication studies, development studies, clinical psychology, and media studies. The paper considers the motivations for people’s decision to watch horror, why people enjoy horror, how individual differences influence responses to, and preference for, horror film, how exposure to horror film changes behavior, how horror film is designed to achieve its effects, why we fear and why we fear specific classes of stimuli, and how liking for horror develops during childhood and adolescence. The literature suggests that (1) low empathy and fearfulness are associated with more enjoyment and desire to watch horror film but that specific dimensions of empathy are better predictors of people’s responses than are others; (2) there is a positive relationship between sensation-seeking and horror enjoyment/preference, but this relationship is not consistent; (3) men and boys prefer to watch, enjoy, and seek our horror more than do women and girls; (4) women are more prone to disgust sensitivity or anxiety than are men, and this may mediate the sex difference in the enjoyment of horror; (5) younger children are afraid of symbolic stimuli, whereas older children become afraid of concrete or realistic stimuli; and (6) in terms of coping with horror, physical coping strategies are more successful in younger children; priming with information about the feared object reduces fear and increases children’s enjoyment of frightening television and film. A number of limitations in the literature is identified, including the multifarious range of horror stimuli used in studies, disparities in methods, small sample sizes, and a lack of research on cross-cultural differences and similarities. Ideas for future research are explored.

## Horror: An Introduction

“It seems an unaccountable pleasure which the spectators of a well-written tragedy receive from sorrow, terror, anxiety and other passions, that are in themselves disagreeable and uneasy” (Hume, 1907).

Why do people watch, and enjoy watching, horror films, and why is this an important or useful question to ask? The primary aims of the horror film are to frighten, shock, horrify, and disgust using a variety of visual and auditory leitmotifs and devices including reference to the supernatural, the abnormal, to mutilation, blood, gore, the infliction of pain, death, deformity, putrefaction, darkness, invasion, mutation, extreme instability, and the unknown (Cherry, 2009; Newman, 2011). It is the emphasis on these characteristics that tend to distinguish horror from the related genre of thriller or psychological thriller (Hanich, 2011). Thrillers are designed to create suspense and terror, but the creation of these feelings is dependent not on the presence of mutilation, gore, or the supernatural but *via* more human devices. These boundaries, however, can be fuzzy. If these features are utilized in thrillers, they are not the principal focus of the film but are incidental to it (an example would be the ear-cutting scene in *Reservoir Dogs,* which is bloody and brutal but is contained within a film, which has a non-horror theme). Together with Westerns, science fiction, comedy, musicals, documentaries, and other film genres, which are characterized by particular tropes, styles, themes, characters, and visual leitmotifs, horror sets itself apart from other film types *via* its distinctive characteristics.

Although commercially successful, the cinematic reputation of horror film has been less than stellar. It has been frequently regarded (if it is regarded at all) as the runt of the cinema family and held in lower esteem than other film categories (Stone, 2016). Etchison (2011) observed that “The horror film occupies in popular culture roughly comparable to that of horror literature. That is to say, it is generally ignored, sometimes acknowledged with bemused tolerance, and viewed with alarm when it irritates authority - rather like a child too spirited to follow the rules that rendition has deemed acceptable” (p. ix), a view that is echoed elsewhere. For example, Tudor (1997) noted that “a taste of horror is a taste for something seemingly abnormal and is therefore deemed to require special attention” (p. 446). Part of the reason for the disdain, apart from the broad and base nature of the content, may be the relative cheapness of horror film: these are often much less expensive to create than are other genre films such as westerns, comedies, or science fiction. The first horror film can probably be dated to 1855/1856. The Lumiere Brothers’ *L’arrive d’un train en gare de la Ciotat* depicts the arrival of a train into a station, the appearance of which, if anecdotal although possibly apocryphal accounts are to be believed, resulted in the audience becoming consumed with a fear that the train would emerge from the screen, such was the novelty of such a depiction at the time.

In terms of industry regard, the reputation of horror has not been high. The American Academy of Motion Picture Arts, which awards the Oscars, has nominated only six horror/supernatural films for Best Picture, and only one has won the Award (*The Silence of The Lambs* in 1992, which also won the award for Best Actress, Actor, and Director). Other horror films to have been nominated include *The Exorcist* (1973), *Jaws* (1975), *The 6th Sense* (1999), *Black Swan* (2010), and *Get Out* (2017). The latter also nominated for best comedy/musical at the Golden Globes and was winner of the Oscar for Best Screenplay. Industry recognition for horror film has tended to be reserved for technical achievements; hence, the Oscars awarded for best art direction and cinematography for *Phantom Of The Opera* (1943), best score for *The Omen* (1976), best visual effects for *Alien* (1979), and best-make up for *An American Werewolf In London* (1981) and *The Fly* (1986). The number of actors to have won an Oscar nomination for horror roles is low – Frederic March (*Dr Jekyll and Mr Hyde*, 1931), Ruth Gordon (*Rosemary’s Baby,* 1968), Kathy Bates (*Misery*, 1990), Natalie Portman (*Black Swan*, 2010), and Hopkins are exceptions.

Despite the relative lack of formal industry recognition and professional respect, horror thrives. In 2017, the second cinema adaptation of the Stephen King novel *IT* (2017) generated $700.4 m in global ticket sales, making this the most financially successful horror film of all time based on recorded box office sales (its production budget was $35 m). The success led to a sequel released in 2019 (*IT: Chapter 2*), which has achieved global ticket sales of $185 m in its first week of release. In 1989, two horror films had grossed over $38 m (*The Fly II* and *The Abyss*, earning $38.9 and $89.8 m, respectively). In 2017, this number was 15, with *IT* leading and occupying 13th place in box office revenue. *The Mummy* occupied 23rd position, *Resident Evil: Final Chapter* the 30th position, *Annabelle: Creation* the 32rd, and *Get Out* the 37th ($255 m). Nine horror films earned more than $100 m in 2017. These numbers illustrate how successful and popular the horror film has become and that viewers’ appetite for it is rapacious.

This commercial enthusiasm exists against a backdrop of considerable fan enthusiasm for the genre, as evidenced by the number of major, significant genre-specific international film festivals which exist. These include the UK’s three Frightfest events, the Sitges International Fantastic Film Festival in Catalonia, Toronto’s After Dark Film Festival, Screamfest and Fantasticfest in the USA, the Brussels International Fantastic Film Festival, Australia’s A Night of Horror International Film Festival, Amsterdam’s Imagine Festival, Argentina’s Rojo Sangre, Italy’s Ravena Nightmare Film Festival, Wales’s Abertoir, and several others. A number of print magazines devoted to horror is available (such as *Rue Morgue, Diabolique, Scream*, and *The Dark Side*) as are various horror websites, online film streaming services (such as *Shudder* and *Screambox*), and specialist satellite/Freeview TV channels such as *The Horror Channel* and *SyFy*. The TV company AMC airs and produces original horror content (and created *Shudder*), and an Asian-based pay-TV horror channel is available called *Thrill*. Given the popularity of horror film, a useful question to explore is why people are attracted to this genre of film, given its distinctive nature, and why people are attracted to horror in the first instance, a question addressed in this review.

Historically, horror has formed a significant part of “Western” literary tradition since the Babylonian *Gilgamesh* and the English *Beowulf*. The Gothic tradition, a period that covers 1,760–1,820 features fiction in which the omphalos is their archaic themes, haunted castles, stylized period settings, a supernatural element in the story telling, suspense, and chaos (Punter, 2014). Examples include Walpole’s *The Castle of Otranto*, Radcliffe’s *Mysteries of Udolpho,* and *The Italian* and Lewis’ *The Monk,* among others. Although modern horror clearly has its roots and traditions in Gothic horror (and castles, spirits, and ghosts are well-documented tropes of horror films), very little modern horror film has been directly inspired by, nor has adapted, these works. Victorian literature has exerted a much greater and direct influence, as evidenced by the re-imaginings and remakes of films based on literary characters from this period, such as Dracula, Frankenstein (doctor and creation), Dr. Jekyll and Mr. Hyde, the Hunchback of Notre Dame, the Phantom of the Opera, Dr. Moreau, Dorian Gray, the monsters and protagonists in Grimm’s Fairy Tales, and the trolls of Nordic literature. These figures have been interpreted and re-interpreted throughout the twentieth and twenty-first centuries in different fictionalized forms – in theater, drama, radio, television, short stories, novels, and, especially, film.

Given the longevity of horror as a genre and its history in cinema, what is it that draws people to this particular genre and how does the genre create the psychological effects that it does? The study of individuals’ response to horror can be illuminating for several reasons. It may help us understand why people are attracted to a very commercially successful genre of film making but one which is seen as very distinctive and highly specialized. It may also help us to explain why some material that is perceived as being unpleasant and disgusting is appealing to some people more than it is to others. The study of horror film may also help us understand how emotions are generated and processed and may help us understand elements of fear (and the attraction of fear).

The current paper sets out to review the literature regarding the appeal of horror and why and how horror cinema exerts the effects that it does. Specifically, it will consider whether there are personality types or other individual differences associated with preference for, and enjoyment of, horror films; whether sex differences exist in the preference for, and enjoyment of, horror film; how fear of horror film develops and how coping strategies are recruited to manage the fear elicited by horror; the psychological and emotional consequences of watching horror and whether watching horror is associated with any adverse, short-term, or long-term psychological consequences; the behavioral responses reliably elicited by exposure to horror film; and the use of auditory stimulation to manipulate our response to horror. A number of texts exists that have discussed and addressed various aspects of horror and horror film, including the cinematic portrayal of the “mad scientist” (Tudor, 1989; Frayling, 2013), the esthetics of horror film (Sipos, 2010), the philosophy of horror (Carroll, 2003), the process of horror fiction writing (King, 2010), the use of sound and music in horror (Hayward, 2009), and the marketing of horror films (Hantke, 2004), among others. To the author’s knowledge, this is the first attempt to assimilate the psychology and related literature in a comprehensive review of our understanding of the enjoyment of horror film, the motivation to watch horror film and the effects of watching horror film. This review was based on keyword searches made *via* Google Scholar and PsycInfo between 2018 and August 2019 and included combinations of the words and terms “horror,” “terror,” “film,” “movie,” “cinema,” “fear,” “thriller,” “slasher,” “fright,” “gore,” “anxiety,” “the unknown,” “the uncanny,” “Gothic,” “blood,” “guts,” “scream/screaming,” “shudder,” shivering,” “trauma,” and “disgust/disgusting.” Material was also sourced from the reference sections of the papers obtained and of books where the topic was horror. The review begins with a definition of horror.

## What is “Horror”?

The word “horror” derives from the Greek *phryke* (meaning “shudder”) and describes the physical manifestations of shivering, shuddering, and piloerection. In the fourth stasimon of Sophocles’ *Oedipus Tyrannus*, the chorus says after the protagonist blinds himself: “Alas, poor man, I cannot ever look at you … such is the shiver (phryke) you cause in me” (Cairns, 2015). An exact and precise modern definition of horror, however, is difficult to determine. Horror has been defined as a “spontaneous response to shocking visual stimulus” (Ceirus, 2015) and as “a compound of terror and revulsion” (Kawin, 2012). In Kawin’s interpretation, “imagined horror provides entry to the made-up world where fears are heightened but can be mastered … it accesses a core of fears we may share as humans, such as the fear of being attacked in the dark … it provides a way to conceptualize, give shape to and deal with the evil and frightening.” Horror, Stone (2016) argues, “confronts us with the disgusting and the fascinating simultaneously,” two aspects of horror returned to later. Horror, according to Marriott (2012), is “the madwoman in the attic.”

One view of horror considers it to be of two types: horror, which is genuine and is designed to make us afraid because it is advantageous to our survival (e.g., fear arising from attack and being motivated to fight or flee), and “art horror,” which describes the imagined horror found in horror films (Carroll, 1987). Carroll also argues that “horror novels, stories, films, plays and so on are marked by the presence of monsters of either a supernatural or sci-fi origin” (p. 52). In Carroll’s definition, it is the presence of a monster, which defines the essence of a horror film, as monsters do not exist within our conventional realm of understanding or reason; they defy science; they should not exist. Carroll views films that are typically classed as horror (e.g., *Psycho*) to be of a different type (tales of terror) because “though eerie and scary, [they] achieve their hair-raising effect by explaining extreme psychological phenomena that are all too human.” This definition, of course, would exclude a significant number of obviously horrific horror films such as *The Silence of the Lambs*, *Henry – Portrait of A Serial Killer*, the *Saw* and *Hostel* franchises and other exemplars of the “torture porn” horror sub-genre and the cannibal films of the 1970s (e.g., *Cannibal Holocaust* and *Cannibal Ferox*). The view has also been challenged (Gaut, 1993). “Slasher” movies, for example, are clearly horror films but do not necessarily contain monsters as described by Carroll (although some, such as Freddie Kruger, Jason Voorhees, and Michael Myers, possess supernatural elements, and Freddie Kruger is an oneiric fiction). Also, Chewbacca and The Force defy the conventions of science, but *Star Wars* would not be classed a horror film.

Horror invariably includes an element of evil, channeled *via* a human, a creature, or a supernatural force, which has the power to change events causing disruption and instability and which must be challenged and defeated (Kjeldgaard-Christiansen, 2016). If this force is not human or supernatural (ghostly, spectral), it is natural – plants, monkeys, ants, leeches, sharks, birds, dogs, bats, rats, bees, fish, earthworms, alligators, spiders, snakes, cockroaches, and dinosaurs have all been employed to create chaos and instability in horror films. Freud (1919/2003) referred to horror as the uncanny (a peculiar translation of “unheimlich, meaning “unhomely”): “the name for everything that ought to have remained secret and hidden but has come to light.” Horror films also invariably present a Manichean view of the world, where good battles evil (as is literally the case in films such as *Dracula, The Exorcist,* and *The Omen*). There is a driving motivation to overcome “a pure and unmotivated desire to inflict suffering” (Clasen, 2014). But horror film, despite the features that the genre shares, is not a unitary cinematic phenomenon and distinct sub-genres or branches exist which are characterized by similar features or styles of film making and storytelling. Often, these are *post hoc* classifications of films, which seem to share core features, and the classification can seem like an exercise in pattern recognition. There are films, which do not easily lend themselves to these classifications (and some may straddle boundaries). However, the most common and typical sub genres include gothic, supernatural/occult/parananormal, psychological horror, monster movies, slasher films, body horror/horror typified by extreme gore, exploitation cinema (Cherry, 2009), and found-footage, which have a very specific technical film-making approach and its own identifiable tropes bequeathed from such films as *Cannibal Holocaust* (1980) but more demonstrably from *The Blair Witch Project* (1999).

Horror film is the only fictional genre, which is specifically created to elicit fear consistently and deliberately rather than sporadically or incidentally. Behaviorally, horror film can create shivering, closing of the eyes, startle, shielding of the eyes, trembling, paralysis, piloerection, withdrawal, heaving, and screaming (Harris et al., 2000). It can produce changes in psychophysiology, specifically increasing heart rate and galvanic skin response (see below). Mentally, it can create anxiety, fear, empathy, and thoughts of disgust (Cantor, 2004). One of the earliest empirical studies to examine the effect of watching horror or suspenseful cinema on behavior asked participants to watch three programs, which varied in suspense (high and low) and in outcome – where the film had a resolved ending or an unresolved ending (Zillmann et al., 1975). The suspenseful programs with the resolved endings were better appreciated than were those with unresolved endings. However, similar – but smaller – results were also found for the unresolved endings (i.e., appreciation levels were high if the program was suspenseful). Cantor (2004) asked students to write about their experiences of horror films and analyzed 3 years’ worth of the students’ papers (530 in total). Approximately 46% of the sample reported experiencing sleep disturbances after the event and 75% reported having experienced anxiety. The four most frequently cited causes of frightening experiences were the films, *Poltergeist* (5.5%), *Jaws* (4.3%), *Blair Witch Project* (4.2%), and *Scream* (3.2%). There were some film-specific anxieties – respondents would express fear of swimming in lakes and oceans, uneasiness around clowns and televisions, and avoidance of camping and woods.

Behavioral change has also been examined experimentally. Hagenaars et al. (2014), for example, asked 50 participants to watch neutral, pleasant, or unpleasant film clips while “standing on a stabilometric platform.” This device measures a person’s motoric behavior as participants engage in some exercise or task. They found that when participants watched unpleasant films, the participants would freeze, show reduced body sway, and heart rate deceleration. The reduced body sway was found early on in the viewing of the unpleasant material (1–2 s after stimulus onset) suggesting that the behavioral effects of watching horror are immediate. The study is one of the few, methodologically well-controlled studies of behavioral response to films designed to elicit strong emotions (pleasant or unpleasant) and demonstrated empirically how exposure to certain types of film affects physical behavior and, in this specific example, how certain types of film inhibit motor behavior.

People’s enjoyment of horror can also be affected by priming. Cantor et al. (1984) found that providing adults with information about the types of events they were about to see in four horror films increased the degree of fright and upset that the participants experienced. Neuendorf and Sparks (1988) extended Cantor et al.’s study by presenting 121 attendees of two horror films (*The Texas Chainsaw Massacre* and *Night of the Living Dead*) at a US cinema with three levels of warning – the low warning involved the transmission of basic information such as the film’s name, the release date, and its R rating; the moderate warning involved all of the low information plus a description of the film’s content; the high warning included both of these plus a statement about a graphic scene in the film (e.g., a paraplegic being sawn in half by a saw-wielding maniac). If individuals reported being previously afraid of the specific types of content mentioned by the experimenters, these “cues” significantly predicted overall fear when prior experience of the film and anxiety was controlled for (fear was measured *via* questionnaire rather than behaviorally). There was no significant correlation between a trait known as sensation seeking (see below) and liking and enjoyment of either film. There was a correlation between prior experience and enjoyment for *The Texas Chainsaw Massacre* suggesting that viewers repeated their viewing because they enjoyed it the first time. Viewers’ anxiety level predicted the fright generated by *Night of the Living Dead*, as did fear cues. The greater the experienced anxiety and the fear cues, the greater the experienced fright. The availability of spoilers – the reveal of key scenes and plot points in a work of fiction in advance of viewing – appears to have little effect on the positive enjoyment of horror film or the experience of suspense (Johnson et al., 2019).

Our behavioral reaction to horror tends to be consistent, although there is not much research that has explicitly investigated this response. The next section considers some of the reasons why people watch horror film and considers some of the dominant theories and models in interdisciplinary research that have been proposed to explain our enjoyment of horror film. It considers first some of the most salient ways in which horror film sets out to frighten viewers including sound.

## Sound in Horror

In addition to the visual and verbal (dialogue) impact of horror, perhaps one of the most significant elements of horror film is auditory. To this end, some authors have argued that “horror is primarily a sound-based medium” (Kawin, 2012): The creaking door, the scream, the shriek of an owl, the hiss of a cat, the squelching of a head as it meets a sledgehammer, the ringing of a phone, the bang of a falling object, and the crack of a branch in an otherwise quiet forest at night are all auditory devices deigned to make viewers and listeners afraid and to create suspense.

One of the most successful, and the most common, auditory tropes in horror is the use of a loud sound after a prolonged period of silence – the so-called “jump scare.” Often the sound is unconnected with what is on screen, but a loud noise might accompany a reveal, such as a face (an example from the genre would be a character opening a mirrored bathroom cupboard door, then closing to discover the reflection of another person standing behind them, with accompanying loud noise or musical note). A distinction is sometimes made between diegetic sound (which the characters can hear) and non-diegetic sounds (which is external to the characters, such as incidental music). Famous examples of the latter are the stabbing and screeching sound of Bernard Herrmann’s violins during the shower scene in *Psycho,* John Williams’s double bass that precedes the appearance of the shark in *Jaws,* John Carpenter’s “stings” and soundtrack in *Halloween,* and the foreboding chorus in *The Omen.* Carpenter has noted that when his film was screened without a soundtrack to a film executive “she wasn’t scared at all. I then became determined to ‘save it with music’” (Hayward, 2009). The high strings and low bass of *Psycho* were influences on Carpenter and Dan Wyman’s score and its 4/5 signature leitmotif, as was the use of Mike Oldfield’s Tubular Bells from the opening of *The Exorcist.*

Some examples of diegetic sounds in horror film include the bangs and creaks caused by entities that are invisible to the actors on screen; one horror film that relies less on gore and blood and more on the potency of audition to increase suspense is *The Blair Witch Project* with its use of nocturnal wails, screams, and creaking branches. The use of sound to amplify horror can be identified in many early horror films – Ruben Mamoulian’s *Dr Jekyll and Mr Hyde* (1931), for example, which includes the first use of the sound of a human heartbeat in film, is familiar for the creation of the “Mamoulian sound stew” of noise, and sound used to generate suspense and excitement in the film.

The second most common auditory influence in horror cinema is the use of music and soundtrack. Research suggests that different styles of music can affect the emotional perception of what is seen in film, regardless of the content (Bullerjahn and Guldenring, 1994), and this accompaniment allows us to interpret what we see in the context of this music (Gorbman, 1987). In horror film, music even has its own trope or leitmotif – the tritone or diabolus in musica (“the devil in music”) otherwise known as the Devil’s tritone (Lerner, 2010) and can be heard in *Beetlejuice, Hocus Pocus* (1993), and *The ‘Burbs* (1989).

Some types of music are designed to be unpleasant, be perceived negatively, and to create tension, and there are many examples of this design in horror film, as discussed earlier. Discordant music has been associated with activity in different brain regions to those found when listening to harmonic or pleasant music; these regions include the right parahippocampal gyrus and precuneus and bilateral orbitofrontal cortex (Blood et al., 1999) and may suggest that these regions are involved in mediating our auditory response to some aspects of horror film. Frightening music has been associated with changes in monoamine receptor activity in the caudate nucleus and right amygdala (decreases) and in the neocortex (increases) in 10 men (Zhang et al., 2012). This study did not include a comparison film clip, however, so the conclusion that can be drawn from it is limited.

The most well-used auditory (and visual) device in horror film is the startle reflex (SR), and this tends to be provoked by the jump scare referred to earlier – the sound of a bump, a sudden burst of noise, some dialogue, or music (Baird, 2000). The first known example of a startle effect in horror film is seen and heard in *The Cat People* (1942) when the sound of a bus door opening occurs just when the viewer is expecting an attack, but the film cuts to this noise and the shot of the door opening. A more recent example can be found in *Fatal Attraction* where a child’s scream and the whistling of the kettle in the reveal of the boiled rabbit overlap. In the same film, Glenn Close’s character’s resurrection in the bath provides another example of the jump scare that employs an auditory device.

Under laboratory conditions, a startle reflex (SR) is produced by delivering 50 ms of 95 db of white noise at unpredictable intervals, while eyeblink is measured. The stimulation is not always auditory and can be visual or tactile. The acoustic startle reflex describes an in involuntary eyeblink, measured at the orbicularis oculi muscle *via* EMG, in response to this noise. The startle reflex can be potentiated when individuals anticipate danger (Grillon et al., 1993a,b; Bublatzky et al., 2013; Bradley et al., 2018) and when pleasant stimuli signal threat (*via* conditioning) (Bradley et al., 2005). This is called affective modulation of the startle reflex, and the startle potentiation is thought to reflect a person’s emotional reactivity to threat. When people watch fear-related or violent films, the blink magnitude (SR) is larger than when people watch films with sexual content (Jansen and Frijda, 1994), neutral content (Koukounas and McCabe, 2001), or sad content (Kreibig et al., 2011). The startle reflex is also greater when people watch unpleasant slides – and smallest when people watch pleasant slides (Vrana et al., 1988) – and when people listen to unpleasant music (Roy et al., 2009). Roy et al.’s study, however, includes a very small sample of 16 participants.

The SR is higher when people recall fear-related sentences than when recalling neutral sentences (Vrana and Lang, 1990) and is higher when people are exposed to negative stimuli than positive or neutral stimuli (Cook et al., 1991), and this is referred to fear-potentiated startle (Grillon et al., 1993a,b). Women’s SR tends to be higher than men’s when stimuli are disgusting (Yartz and Hawk, 2002). Fear, however, is the stimulus that creates the greatest SR (Bradley et al., 1999) and people with specific phobias show potentiated SR when phobia-related stimuli are viewed. Some studies find that a SR does not occur to some types of negative stimuli such as mutilation or surgery (Stanley and Knight, 2004). The startle effect is a highly replicable behavioral phenomenon and can be reduced with the administration on anxiolytics and when lesions are made to the amygdala (Hitchcock and Davis, 1986; Angrilli et al., 1996; Davis, 2006). It would be instructive to study whether those high and low in empathy or sensation seeking (see below) and whether individuals who like horror film and those who dislike horror film would generate different SRs.

## Why do People Watch Horror?

Suspense and resolution of suspense are two important components of horror and our response to horror film. Suspense refers to the build up to threat, the tension created prior to the manifestation of threat, and the resolution/elimination of threat. It has been defined as “acute, fearful apprehension about deplorable events that threatens liked protagonists” and “an experience of uncertainty whose hedonic properties can vary from noxious to pleasant” (Zillmann, 1996, p. 108). The tension created during the feeling of suspense can arise from events, which signify conflict, dissonance, and instability (Lehne and Koelsch, 2015). One theory of horror enjoyment, Zillmann’s (1980, 1996) excitation transfer theory, argues that we derive our enjoyment of horror film from this feeling of suspense (this theory might also explain the enjoyment of non-horror film, which involves the invocation of suspense). When a threat is resolved, our negative affect converts to euphoria and suspense ends. The vital aspect of the theory is that enjoyment is derived from the degree of negative affect built up during exposure to the horror film and from the positive affect/reaction that results from the resolution of the threat. If the resolution does not occur, then residual negative affect will lead to increased dysphoria. If there is no suspense but a complete certainty about what will happen, suspense is replaced by dread (Oliver, 1993a,b). Very few studies have tested the theory, although limited reviews provide some support for the model (Hoffner and Levine, 2005). Zillmann et al. (1975) showed children animated cartoons that varied in suspense and measured participants’ facial expressions, physiological arousal, and cognitive responses. They found that liking of the film increased as suspense increased. Liking was especially great when the threat was overcome, but the relationship between fear and liking was not examined in the study.

Individuals high in empathy will express more negative affect regardless of a successful resolution to the threat in the film (Zillmann et al., 1986; Hoffner and Cantor, 1991; Sparks, 1991). Zillmann’s model has some difficulty accounting for the motivation to watch and for the enjoyment derived from horror films in which the sympathetic characters are (1) dispatched and (2) where the story does not end happily (Hoffner and Levine, 2005). There is also evidence that enjoyment of horror may not be affected by the availability of resolution and that unresolved horror is perceived as just as enjoyable as resolved horror (Hoffner and Cantor, 1991).

An alternative model to Zillmann’s suggests that enjoyment is associated with the presence of destruction, excitement, and unpredictability in films (Sparks, 1986a,b; Tamborini et al., 1987; Tamborini and Stiff, 1987). This model, the uses and gratification theory of film consumption (Katz et al., 1973; Palmgreen, 1984), argues that the enjoyment and seeking out of material are determined by their specific need for stimulation and the satisfaction they derive following the achievement of gratification. Some research suggests that certain personality types and individuals who are high or low on some psychological traits may seek out horror or violent material for gratification but that the material itself may not always provide this satisfaction (see the Individual Differences section below). Sensation seeking, verbal aggression, and argumentativeness, for example, have been found to be positively correlated with enjoyment of horror and violent films, but these are not consistent predictors of liking for horror/violent material (Greene and Krcmar, 2005).

Zillmann (1980) has argued that a positive outcome for the protagonist and a poor one for the antagonist are the key predictors of satisfaction with a film. If neither occurs but a threat is removed, this would also lead to a satisfactory experience, but the experience would be diluted. A positive outcome is, however, necessary for the “cognitive switch from dysphoria to euphoria” (p. 148). There is no consistent evidence to support this view and the success of films where the threat is still very much present in some way at the end of a horror film (e.g., *The Exorcist, The Omen, Friday the 13th,* and so on) and even in thrillers such as *Basic Instinct* and *Presumed Innocent,* suggests that this explanation may not account fully for why we watch and enjoy horror.

It has been proposed that arousal itself might be self-rewarding – the act of watching horror provides us with a thrill regardless of the resolution and we like and enjoy the film for this reason (Tamborini, 1991). The pleasurable experience of arousal motivates us to continue watching in order to sustain that level of arousal, as Berlyne (1967) suggests. Sparks and Spirek (1988), for example, found a positive correlation between skin conductance (a physiological measure of emotional arousal) and self-reported arousal in people who watched a clip of *A Nightmare On Elm Street*, suggesting that the arousal we report also correlates at the physiological level, although whether the psychophysiological changes determine the arousal or the cognitive and emotional arousals (the interpretation of the material) determine the psychophysiological changes is an argument, which dates back to James.

## Individual Differences in Response to Horror

Carroll (2003) asked, “How can horror audiences find pleasure in what by nature is distressful and unpleasant?” Some research has attempted to answer this question by studying the type of individual who enjoys and likes horror. Some of the personality traits and cognitive/affective traits that have been implicated in horror preference and/or enjoyment of horror include sensation seeking, empathy, theory of mind, need for affect, the dark tetrad, and personality. Other individual differences include age and sex (considered later). Unless a person expresses an interest and liking of horror, the response to graphic violence tends not to be positive. Weaver and Wilson (2009), for example, assigned 400 people to one of three groups who watched either clips from five television programs showing graphic violence, clips with the violence sanitized, or clips with the violence removed. The non-violent programs were regarded as more enjoyable than the violent versions, a finding which is consistent with earlier research indicating that removing the violent content from a film does not reduce the film’s enjoyment (Sparks et al., 2005). A meta-analysis of the enjoyment of media violence (not horror film specifically) found that greater selective exposure to violence (i.e., choosing to watch violent media) leads to a reduction in the enjoyment of its content (Weaver, 2011). The implication of this finding appears to be that even though individuals may seek out exposure to violent media, they do not often enjoy what they find. In addition, participants may vary according to the degree of material they are routinely exposed to. When graduate nursing students and psychology students were shown videos of graphic medical procedures, for example, the nurses expressed less disgust and fear but more sadness (Vlahou et al., 2011). Both groups, however, showed evidence of psychophysiological arousal (measured *via* Galvanic Skin Response) in response to watching the procedures.

### Sensation Seeking

The most widely studied trait in the research on horror is sensation seeking. According to Zuckerman (1994), sensation seeking is the “seeking of varied, novel, complex and intense sensations and experiences, and the willingness to take physical, social, legal and financial risks for the sake of such experiences” (p. 27). It peaks in the teenage years and declines thereafter (Zuckerman, 1988). Zuckerman’s measure of sensation seeking describes four related but different factors: (1) thrill and adventure seeking; (2) experience seeking; (3) disinhibition; and (4) boredom susceptibility. In the original conception of the model (Zuckerman, 1979), individuals thought to be high sensation seekers would experience much more positive emotion when highly aroused and stimulated and would seek negative stimulation to maximize their arousal because this stimulation was intense. A negative stimulus (such as a horror film) might, therefore, be interpreted by a person high in sensation seeking as being very positive; but a person low in sensation-seeking would find the stimulus unpleasant. High sensation-seeking individuals would also be less vulnerable to the experience of threat in these films (Franken et al., 1992).

All four factors of the sensation-seeking scale have been found to predict enjoyment of horror film to some extent, but some factors are better predictors than others. For example, disinhibition was found by Edwards (1984) to be the strongest predictor, followed by experience seeking, thrill and adventure seeking, and boredom susceptibility. Edwards reported a positive correlation between high sensation seeking (in general) and interest in horror film. Tamborini and Stiff (1987) found a positive correlation between liking for horror and a combination of the sensation-seeking factors. Zuckerman and Litle (1986) found that frequency of horror film attendance correlated with disinhibition, thrill and adventure seeking, and boredom susceptibility, but in men only. The sex difference in this study highlights an important constraint on the model, and that is, individual differences (such as sex) may interact with sensation-seeking type to predict viewing, preference for, or enjoyment of horror film (see below). Cantor and Sparks (1984) found that sensation seeking was positively correlated with the enjoyment of frightening films in men and women. However, components of sensation seeking predicted enjoyment differently – thrill and adventure seeking were the best predictor for men, whereas disinhibition was the best predictor for women.

Other studies have reported no positive correlation between sensation seeking and liking and enjoyment for horror films (Neuendorf and Sparks, 1988). Aluja-Fabregat (2000) found that disinhibition and psychopathy – a personality trait which describes a charming, remorseless, callous, and manipulative personality type – correlated with curiosity about morbid events in 470 eighth graders in Catalan. Sensation seeking correlated with consumption of violent films and consumption was associated with psychopathy, specifically in boys.

In a study of the enjoyment of fear experiences in video gaming, Lynch and Martins (2015) found that in their sample of 269 18–24-year-old players, men reported more enjoyment of violent video games and played more games and played more often. Sensation seeking and enjoyment were positively correlated, with high sensation seekers reporting less frequent fear (although *p* = 0.05) and low empathizers enjoying the violent games more. Low empathizers also played more but did not play more frequently. *Resident Evil* was the most commonly played game, and the game’s inclusion of zombies and surprises was cited as a cause of fear and fright. Agency in such games, however, appears to be important to the experience of the medium. When players were either asked to watch or to play a horror computer game (Konami’s “*PT*”), players showed increased heart rate and galvanic skin response (emotional arousal) compared to participants who watched (Madsen, 2016). There were no differences between the two groups in self-reported fear.

While sensation seeking might be strongly associated with enjoyment of horror, it may not be the strongest predictor of attendance at horror films. Tamborini and Stiff’s (1987) study of 155 people (78 men; average age 21 years) attending a horror film in a US Midwestern city reported that men and younger participants scored the highest on the sensation-seeking scale, but that men and women attended for different reasons: men attended because they sought sensation and to experience the destructive nature of the horror while women attended because of they wanted to experience a just ending. More important than sensation seeking appeared to be participants’ expectations of the film: The greatest predictor of film attendance was not sensation seeking but a desire to experience a satisfying resolution (especially by women) and to see destruction (men).

Also of note is that there is evidence that sensation seeking is related to the startle potentiation described earlier. Lissek and Powers (2003) found that people low in sensation seeking (as measured *via* the thrill and adventure-seeking subscale) produced the typical startle potentiation during the viewing of threatening (vs. neutral) images but that those high in sensation seeking showed equal levels of startle to neutral and threatening images. One explanation for this finding is that high levels of sensation seeking are related to low levels of reactivity to threatening images. Because high sensation seeking involves a degree of sensory overload, less stimulation is required for a startle potentiation to occur and those scoring high in sensation seeking show less fear startle potentiation.

The literature on sensation seeking, therefore, suggests that this trait and specific components of it, especially disinhibition, may predict enjoyment of horror film, but this prediction does not apply to men and women consistently (a conclusion considered in more detail in the section on sex differences below). The literature also highlights a limitation in this – and other areas – of the horror research literature in that samples are often heterogeneous, the film selections are heterogeneous, and sample sizes tend to be small. These limitations are returned to at the end of the review.

### Empathy

Empathy is a multidimensional concept whose components have been defined in different ways but which in general are reflected in two types: a cognitive component (e.g., perspective taking) and an affective/emotional component (sympathy and concern for others and sharing of negative affect). One model suggests that empathy comprises a wandering imagination (a tendency to fantasize and daydream about fictional situations), fictional involvement (transposition of oneself into a story), humanistic mentation (a sensitivity to the emotional welfare of others), and emotional contagion (a susceptibility to be influenced by the emotions around oneself) (Tamborini et al., 1990). Zillmann has proposed a three-factor model of empathy in which emotional behavior arises from the interaction of between these dispositional (a “response-guiding” mechanisms, which result in motor reactions to a stimulus), excitatory (“response-energizing” mechanism, which enables immediate arousal), and experiential (the conscious experience of the first two). Davis (1983), who originally developed the Interpersonal Reactivity Index, argued that empathy was not a unitary or binary concept but was best considered as a set of constructs, which involve our reactions to others but are distinct from each other. These constructs included perspective taking, a fantasy scale (which measures the degree to which a person transposes themselves into the feelings or actions of fictional characters), empathetic concern (which measures the degree of sympathy felt for others), and personal distress (a description of unease or distress experienced in interpersonal relationships).

There is evidence that each component can predict enjoyment of horror film, with low empathy consistently associated with greater enjoyment. In one study (Tamborini et al., 1990), 95 young people in same-sex pairs watched clips from two 1-h documentaries or two full length horror films (*A Nightmare on Elm Street* and *Boogens*). The study found that tendency to daydream and fantasize predicted the ability to sense the feelings and actions of the films’ characters. Those scoring high on the wandering item, fictional involvement, humanistic mentation, and contagion scales described above found graphic horror less appealing. Those scoring low in empathy preferred graphic horror. People low in fearfulness also prefer graphic horror (Mundorf et al., 1989). Hall and Bracken’s (2011) study of 199 undergraduates found that fantasy empathy (but no other type) predicted narrative transportation (immersion in a text/film or “getting lost” in a story) and was associated with increased enjoyment of the film, although not necessarily horror film exclusively.

In a variant of this procedure, Tamborini et al. (1993) asked participants to watch a pleasant (a comedy) or an unpleasant (*Videodrome*) film, with a confederate. To evoke empathy, after the film, the confederate said they were distressed because they thought they were going to be thrown out of school and asked “what am I going to do?” If there was no reply, the confederate left. If they received a reply, the responses would be rejected. Those participants scoring high in fictional involvement and empathetic concern provided more comfort and more social support. Those who watched the horror film, however, provided less support than did those who watched the comedy. While providing a potentially useful contribution to the study of how people respond to horror and the effect of this on our interaction with others – the greater the empathy, the greater the responsiveness to others’ distress – the sample size is small (*N* = 21).

Empathy has also been associated with less enjoyment of suffering displayed in frightening films but with more enjoyment of danger, of excitement, and of happy endings (Hoffner, 2009). People high in enduring negative affect have been found to experience more distress and less enjoyment of suffering. Those who had prior exposure to frightening films enjoyed danger more and enjoyed happy endings less.

Classifying participants according to the degree of empathy and sensation seeking has not been the only approach that has been taken to determining the types of people who watch and enjoy/prefer horror. Johnston (1995), for example, notes that not all audiences respond to horror in the same way, as this section has demonstrated and has typologized viewers and their motivations to watch into three types: (1) resolved-ending types; (2) thrill watchers; and (3) gore watchers. Resolved-ending types enjoy film with a satisfying, definite closure; thrill watchers enjoy being frightened and empathize with the principal characters; gore watchers watch because they enjoy the destructiveness in film. The typology is based on some of the research reviewed here. A prediction that can be made from this typology is that thrill watchers will have higher levels of empathy and adventure seeking, whereas gore watchers will be low in empathy and fearfulness but will be high in adventure seeking and will seek out high arousal (King and Hourani, 2007). Research suggests that gore watchers are curious about the ways people are killed, are vindictive (they require satisfaction that characters receive their just desserts), and are attracted to blood and guts (gore) in film (King and Hourani, 2007). Gore watchers are more likely to be men, to identify with the killer in films and are less likely to identify with the victim.

King and Hourani identified types of watchers from 229 individuals and showed them four horror films. Half the sample saw the films with a traditional ending (in which the evil antagonist is destroyed) or with teaser endings (in which the evil antagonist is revived/resurrected). The traditional ending was more entertaining than was the teaser ending, but it was especially enjoyable and entertaining for high gore and thrill watchers than low gore and thrill watchers. Traditional endings were less distressing and more frightening for high than low gore watchers and were regarded as being more frightening by high thrill watchers. High thrill watchers found the teaser ending version of the film to be less scary than did low thrill watchers. High gore watchers regarded the teaser to be more predictable than did low thrill watchers. The traditional ending was considered to be less predictable by high gore watchers than by high thrill watchers and by high thrill watchers than by low thrill watchers. Very little research exists on this typology, however.

Although individual studies indicate a relationship between empathy and horror enjoyment, a meta-analysis of studies investigating the enjoyment of mediated fright and violence has found that empathetic concern and personal distress were negatively correlated with enjoyment, but correlations for personal distress were not consistent (Hoffner and Levine, 2005). The authors note that the inconsistencies may be attributable to differences in the content of the film employed in these studies, and this is a problematic issue common to the field: There are no consistently chosen materials in either nature, content, length, age, or narrative. What is noteworthy, however, is that Hoffner and Levine’s review found that the strongest effects (reported in two studies) were for studies, which included horror films, and those films depicted torture (Johnston, 1995) or brutal horror with no positive resolution (Tamborini et al., 1990). When these studies were removed, the correlation between empathy and enjoyment became non-significant. The authors note that the other four films measured participants’ enjoyment of horror film as a genre (rather than their enjoyment of specific horror films or acts of graphic violence), and this methodological limitation in the literature is returned to the conclusion of this paper.

### Need for Affect

A different form of individual difference – need for affect – may also mediate horror film preference and enjoyment, but the literature is limited. Need for affect (Maio and Esses, 2001) is based on the assumption that we are motivated to seek interesting or positive experiences and avoid unpleasant ones. Need for Affect (NfA) is measured *via* a questionnaire, which comprises two subscales: the tendency to approach and the tendency to withdraw. People who prefer sad films experience more enjoyment when watching sad films, for example, because they regard viewing sad films as an enjoyable and a gratifying experience; their need for affect is satisfied by watching sad films (Oliver, 1993a,b; Oliver et al., 2000; Maio and Esses, 2001). Few studies have explored the relationship between NfA and horror film viewing. One study asked 119 attendees (mean age = 23 years) at a German cinema how likely they would be to watch *United* 93 or the 2006 horror film remake, *The Omen* (Bartsch et al., 2010). Participants with higher NfA approach scores experienced more intense emotions and experienced more negative emotions such as anger, fear, and disgust. *United* 93 evoked more negative emotions than did *The Omen*. Higher NfA withdraw scores were associated with a more negative evaluation of emotions. Controlling for personality did not affect these results significantly. While NfA is little studied in horror, one possible research question that could be explored is whether preference for film genres correlates with NfA; no study to date has systematically examined this relationship.

### Other Personality Traits

Other personality traits thought to be implicated in horror film preference or enjoyment include the Big Five, the Dark Tetrad, and repressive coping style. Dark personality traits are those which express some abnormal, sinister, and unpleasant aspect of behavior. Four such traits are Machiavellianism, Narcissism, Psychopathy (described earlier), and Sadism. Machiavellianism (the enjoyment of power and the manipulation of power) has been found to correlate with enjoyment of horror, and the correlations between these two variables are stronger than the correlation between Machiavellianism and sensation seeking (Tamborini and Stiff, 1987). High psychopathy scores have been associated with preference for graphically violent horror movies (Weaver, 1991), and individuals scoring high in callousness and who habitually express little or no emotion show reduced facial expressions of sadness and disgust when watching violent films (Fanti et al., 2017).

A repressive coping style is characterized by the repression of negative affect caused by stressors (Weinberger, 1990; Sparks et al., 1999). Sparks et al. investigated repressive coping style and enjoyment of horror film stimuli in 59 individuals. Based on a median split, 30 repressors and 29 non-repressors were identified and were asked to view a 25-min extract from *When A Stranger Calls* (in which a babysitter receives frightening phone calls and discovers that the calls have been coming from inside the house she is in). Women in general expressed greater negative affect than did men, as expected (see section below), but the repressors in general showed greater physiological arousal during the film than did non-repressors. An interesting pattern emerged across the course of exposure. Physiological arousal was similar for both groups at the beginning of the first two sections of the movie and then diverged in the final three sections as the suspense increased. No explicit analysis was provided of the psychometric response to the film (how much it was liked, how frightening it was, and so on). The study suggests that those who repress negative affect may nonetheless show high levels of physiological arousal during exposure to frightening films. What is less clear in this study is the relationship between this phenomenon and enjoyment of the film. It is also based on a very low sample of participants, and little subsequent research has focused on this particular personality trait/style.

Despite being the most commonly accepted model of personality, the Big Five has been the focus of very little published research in the context of horror film enjoyment and consumption. The Big Five proposes that personality is comprised of five core traits along which individuals differ. These traits are Conscientiousness, Openness to Experience, Extraversion, Neuroticism, and Agreeableness. One study employing a version of the Big Five found that a trait described as Intellect/Imagination (defined as a proclivity to engage in imaginative activity) was the strongest predictor of horror media consumption (Clasen et al., 2019). There was a small but statistically significant and positive correlation between extraversion and frequency of horror media use, using horror media with others, enjoying horror media with others and being more scared with others. Agreeableness was positively correlated with being easily scared by horror media, using horror media with others, enjoying horror media with others, and negatively correlated with being more scared with others. People high in conscientiousness were less scared after using horror media, and people high in emotional stability were found to be less easily scared than those low in emotional stability, a finding which was also reported by Reynaud et al. (2012), who found that psychophysiological arousal was greater in participants who were high in neuroticism when they watched a film designed to elicit fear. The number of participants in Reynaud et al.’s study, however, was small.

The finding regarding agreeableness contrasts with research on violent video game playing where people lower in agreeableness have been found to be more frequent violent video games players; individuals who score high in extraversion and openness and low in neuroticism have also been found to be more frequent users (Chory and Goodboy, 2011). Low agreeableness is a significant predictor of enjoyment of the horror film genre but not exclusively – it is also a significant predictor of enjoyment of parody, animation, neo-noir, and cult genres across different media including books, television, and film (Cantador et al., 2013). While the findings of Chory and Goodboy (2011) are informative, they are limited in terms of the measurement of response to horror film specifically because the stimuli used were not specifically horror film. A similar limitation can be found in Clasen et al.’s (2019) large Mechanical Turk study of 1,070 participants which asked participants for their responses to and perceptions of horror media generally, not horror film specifically. The study also administered a variant of the Big Five personality inventory and a variant of the sensation-seeking scale (Hoyle et al.’s (2002, Brief Sensation Seeking Scale) not normally administered in research examining the relationship between personality and horror film. Although research on violent video games might help understand some of the correlates between use frequency and personality trait, it should be acknowledged that violent video games are qualitatively different stimuli to films. Films are a passive experience – viewers are unable to influence the action they see on screen – whereas gaming is specifically an active experience where the player engages with what they see and are expected to do so as this is the principal motivation for gaming. Horror films and horror games are not equivalent stimuli, although they share many characteristics and elements of content.

In conclusion, the literature studying the relationship between personality and horror film consumption has been limited in number and scope. Two studies have reported a correlation between low agreeableness and preference/enjoyment of horror media, and one has not. It is noteworthy that in one of the studies reporting an association, agreeableness was the only trait to be significantly associated with horror media use. This aspect of personality may be worth exploring further.

### Sex Differences

The most consistent individual difference predicting individuals’ response to horror film is biological sex: men and boys enjoy frightening and violent visual material more than do women and girls (Zuckerman and Litle, 1986; Harris et al., 2000; Hoffner and Levine, 2005). Correlations between intensity of “scary media” or horror and the enjoyment of horror in men are consistently positive (Hoffner and Levine, 2005). Men enjoy horror media more than do women, are less scared by horror media, use horror media more, and show a greater preference for frightening horror media (Clasen et al., 2019). One of the earliest experimental studies of sex differences investigated the role of social comparison in individuals’ response to horror. Zillmann et al. (1986) asked 36 men and 36 female undergraduates to watch horror films (*Nightmares*, *Nightmare on Elm Street*) in the presence of a same-age, opposite-sex companion who either expressed control, indifference, or distress during the film. Men enjoyed the horror more and found it less boring and more satisfying and frightening than did women. Men expressed more distress if the female companion expressed distress (but engaged more with them than with a masterful woman) and less if the female companion was masterful. Zillmann et al. also manipulated initial appeal of the companion (high and low). Women enjoyed the films more in the company of a man with high appeal, but women’s appeal had little effect on men’s responses. Women engaged more with masterful than with distressed men. Cutting violence from films can increase enjoyability and decrease arousal in women (but has no effect on men): women regard these films to be generally more disturbing than do men (Berry et al., 1999).

Male undergraduates experience less distress and anxiety than do women when watching horror film (Sparks, 1991), and women find film clips depicting sadness and fear more unpleasant and distressing; they also show greater arousal to fear clips than to clips depicting compassion (Davydov et al., 2013; Maffei et al., 2015). The findings reflect a more general sex difference in that women, in general, report greater fear and anxiety than do men. Women have been found to express more fears, more severe fears, and greater fear of repulsive but harmless animals (Tucker and Bond, 1997), a finding that applies cross-culturally (Arrindell et al., 2004). Anxiety disorders are more commonly reported by women than men (McLean and Anderson, 2009), and women appear to be more susceptible to variety of anxiety-related disorders such as panic disorder, generalized anxiety disorder, PTSD, and agoraphobia (Kessler et al., 1994). The exception to this pattern is fear of bodily injury, social stimuli, noise, or enclosed spaces, where no consistent sex differences have been reported (McLean and Anderson, 2009). Disgust sensitivity – the degree to which individuals find stimuli repulsive – also tends to be higher in women, and this phenomenon might provide an explanation for the sex difference in the fear of animals – and horror film (Connolly et al., 2008). This is considered in more detail below. Women and girls, for example, are less likely to enjoy violent media when blood and gore portrayed are described as extreme, rather than mild or moderate (Hoffner and Levine, 2005).

The sex difference is not only reported in the horror genre but also across a number of cinematic genres. One study of 150 undergraduates in Germany (Wühr et al., 2017) asked participants to indicate which types of films they believed that men and women would generally prefer. In a second study, participants were asked to indicate the films they themselves preferred. In the first study, men were regarded as preferring action, adventure, erotic, fantasy, historical, horror, sci-fi, thriller, war, and western films, whereas women preferred animation, comedy, drama, heimat, and romantic films. Both sexes liked crime and mystery equally. In the second study, women expressed a preference for drama and romance, and men preferred action, adventure, erotic, fantasy, horror, mystery, sci-fi, war, and Western films. Animation, comedy, crime heist, history, and thrillers were liked by both sexes.

Enjoyment and liking of the degree of explicit (graphic) horror also appear to show sex differences. Men tend to prefer very graphic horror material more than do women (Hoffner and Levine, 2005). Men also report watching more violent television and attend more horror films. One explanation for this finding has been proposed by gender socialization theory (Zaslow and Hayes, 1986), whereby boys and men are socialized to not be afraid and to not make expressive shows of fear, whereas girls are not constrained by such expectations and can “express their sensitivity by being appropriately disturbed” (Hoffner and Levine, 2005). Such an explanation is probably locked in a prison of its own time in the sense that it is unclear whether such attitudes still exist now, at the end of the second decade of the twenty-first century.

Sex differences have been reported in the context of other behaviors such as the identification with a film’s character. Tamborini et al. (1987) asked 44 male and 50 female undergraduates to rank their preference for two different versions of 13 films (12 of which were fictional). In one version, the victim of graphic violence was male; in the other, the victim was female. One theory of horror enjoyment discussed earlier (the uses and gratification perspective; Rubin, 1994) argues that our reasons for watching horror and the benefit and gratification we derive from it will determine whether we identify with a victim or an aggressor (Johnston, 1995). Viewers who identify with a female victim are usually more likely to experience distress (Zillmann and Cantor, 1977) and are not satisfied by happy endings (Tannenbaum and Gaer, 1965). Oliver’s (1993a,b) study of 96 16-year-old high school students found that there was a correlation between gore watchers and enjoyment of retribution (liking to see victims get what they deserve). Participants’ high punitive sexual attitudes have been found to be positively correlated with higher ratings of enjoyment; men prefer horror films in which the female rather than the male is the victim, but there is no significant association between enjoyment and the films’ portrayal of victimization of sexual characters, of women, or of women expressing their sexuality.

Tamborini et al. (1987) found that participants’ recent and past viewing of horror film strongly predicted enjoyment of graphic horror in general. However, the responses to men and women as victims in the film interacted with other viewing preferences. For example, men’s enjoyment of pornography was correlated with preference for graphic horror, which depicted female victimization but not male victimization. Preference for graphic horror correlated with disinhibition, moderately for boredom susceptibility and experience seeking, and not at all for thrill/adventure seeking. Sensation seeking in general did not predict preference for graphic horror. Women regarded the films with female victims to be higher in violent content than films featuring male victims; the opposite pattern was found in men. Boredom susceptibility was a good predictor of preference for graphic horror in men. No one factor was a strong predictor of graphic horror preference in women when the victim was male. Deceit and boredom susceptibility predicted graphic horror preference when the victim was female. Physiological arousal (measured *via* GSR) has also been correlated with enjoyment of horror after men finish watching a film (Sparks, 1991).

A retrospective study of 233 psychology students (125 men) asked participants to recall details of a date they had been on as a teenager/young adult during which they watched a frightening film (Harris et al., 2000). The participants reported that the films most commonly seen were *Scream, Scream 2*, *I Know What You Did Last Summer,* and *I Still Know What You Did Last Summer*. Men were younger when they watched the film (16.7 vs. 17.6 years), and the study found some notable and significant sex differences: Thirty-one percent of women reported looking away from the screen, whereas only 7% of men did. About 61% of women reported feeling anxious, whereas 44% of men did; 34% women reported that it had increased their imagination (men – 1%); 19% of women said they feared sleeping alone afterward (men – 8%); 67% of women said their heartbeat were faster (men – 53%); 56% of women said they became very jumpy (men – 31%); 41% of women were amused and entertained (men – 59%); 55% of women held onto their date (men – 21%); 32% of women screamed (men – 6%); and 26% of women felt disgusted (men – 10%). Men gave more positive reactions than did women, and women gave more negative reactions than did men, and women reported more sleep disturbances than did men. About 80% of women reported being somewhat or very afraid (men – 46%), and 18% reported not being afraid or being a little afraid (men – 51%). This study also measured empathy and found a positive correlation between overall empathy scale scores and negative reactions but not between negative reactions and any one specific subscale. There were some associations between negative reactions and empathetic responses. Low empathetic concern, for example, predicted sleep disturbance. Higher boredom susceptibility was associated with fewer negative reactions and with increased liking but not with sleep disturbance. Women who scored high on empathy were more likely to be scared at the time of the study (i.e., they were more likely to express fear as adults) than were low-scoring women or men generally.

In a similar study, Hoekstra et al. (1999) asked 202 introductory psychology students to describe their reactions (especially fear reactions) when they recalled the frightening movies they watched as children. The mean age at watching was 10.8 years, a similar finding to Cantor (2004). Female participants as adults liked slasher films less than did male participants as adults – of the 14 categories included, this was the least liked by women. The most liked genre by women was romantic comedy; by men, action and adventure. Men reported choosing to watch horror more often than did women. Both sexes noted fear-related changes after watching films as children but not during the film, with women reporting more negative reactions during the watching of the films when they were girls. The earlier their exposure to horror films as children, the greater was the sleeping disturbance they experienced afterward. The behavioral measures indicated the typical sex differences reported earlier: more girls than men hid their eyes (64 vs. 26%), held someone (35 vs. 6%), and were jumpy (65 vs. 45%).

In terms of the enjoyment of specific content, one study asked participants to rate a 10-min horror film in which the sex of the victim and sexual content was manipulated (Oliver, 1994). The context of this study concerned the types of victim and protagonist in slasher films. An earlier content analysis of 10 slasher films found that a third of sex scenes concluded with the death of a character (Weaver, 1991). Women, however, are not more likely to be killed. In an analysis of 56 slasher films, Cowan and O’Brien (1990) found that men and women were equally likely to be killed off. Women were more likely to be survivors, a cliche that has its own term in horror film: the Final Girl. More screen time is devoted to the deaths of women than men, however, and non-surviving women are more likely to be promiscuous, wear revealing clothes, appear nude, use sexual language, and undress and engage in sex when they are killed. Non-surviving men appear to be identified only by their use of sexual language. Oliver (1994) found that sexual portrayals of victims were associated with increased viewer enjoyment, especially in men. These films were also regarded as more frightening.

As discussed earlier, one possible explanation for women’s reaction to horror may be their disgust sensitivity. Women in general report greater disgust sensitivity than do men. Disgust is a protective response to a direct threat to survival, such as contamination, lesions, sores, or disease (Krusemark and Li, 2011). People high in disgust sensitivity show higher levels of disgust toward low, moderate, and severe facial disfigurement (Shanmugarajah et al., 2012). Individuals with anxiety disorders are more prone to be disgusted, especially those who are anxious about contagion (Olatunji et al., 2017a,b). People who are exposed to disease primes are more likely to judge themselves to be less extravert and open to experience (Mortensen et al., 2010), and people distance themselves from contagion or symptoms of contagion (Neuberg et al., 2011). Women’s disgust thresholds for imagining incest, reacting to images of insects, seeing open sores, feces or dirty clothing, and statements about death and sex are significantly lower than those for men, and women are less likely to work in environments in which pathogen exposure is likely (Al-Shawaf et al., 2018). Women’s sexual disgust and pathogen disgust are higher than that for men, but their moral disgust appears to be no difference. This elevated sense of disgust sensitivity in women may partly explain why they enjoy horror film less than do men.

The literature on sex differences in response to, and preference for, horror film provides the most consistent finding in the field that men and boys prefer and enjoy horror film more than do girls and women. One possible explanation for this, besides differences in empathy, may lie in differences in higher-order traits such as anxiety proneness and disgust sensitivity. This possibility, and the evidence for it, is discussed in a later section.

## Horror Films and Mental Health

While a typical person’s response to horror film is fear and anxiety, some studies have suggested that exposure to horror films can lead to abnormal stress or distress reactions requiring psychological or psychiatric intervention, a condition called cinematic neurosis (Ballon and Leszcz, 2007). The rarity of these case studies and the details they present – Ballon and Leszcz found only seven such case studies – suggests that the individuals’ behavior arise because of causes unrelated to the horror film and that the horror film was a catalyst for provoking an underlying and pre-existing pathology that would have been provoked by any, other relevant stimuli. The pattern of behavior has echoes in Freud’s (1919/1971) account of seventeenth century “demonological neurosis,” whereby depression or psychosis arose from experiencing the death of a father and individuals made a pact with the Devil to relieve their distress.

According to Johnson (1980), at least a quarter of horror film viewers had experienced “stress-type” reactions, although this is likely to be within the confines of the normal stress reaction that horror is specifically designed to evoke. Many of the studies reported are case studies, lacking in control participants and largely anecdotal. In a typical example, Horowitz and Wilner (1976) observed that after the release of *The Exorcist* in 1973, individuals lost “control over thought and emotions,” experiencing “denial and numbing … extremes of anxiety, tension and impaired relationships.” *The Exorcist* is the source of a number of abnormal behaviors reported by individuals responding extremely to horror film.

Bozzuto (1975) described four adults who developed abnormal stress behavior within a day of watching the film; participants reported insomnia, excitability, hyperactivity, irritability, and decreased appetite. The symptoms dissipated after seven sessions of psychotherapy. Mathai (1983) reported the case of a distressed 12-year-old boy who felt that when somebody touched him, they would go right through him and that when sitting on a chair, he would fall through it. Prior to presentation, he had watched *The Invasion Of The Body Snatchers* with two of his siblings. Waking from his sleep, he saw “an awful face with bulging veins staring at him.” Hamilton (1978) reported the case of a young woman who had seen *The Exorcist* and presented with “acute unremitting anxiety and a pervasive fear of being alone especially at night” and refused to go to work. She felt that the “Devil was in a young girl” and “she dreamt of the Devil with a penis in his mouth” (p. 569).

Five of the cases identified by Ballon and Leszcz (2007) cited *The Exorcist* as the cause of their distress. The other two were *Jaws* and *Invasion Of The Body Snatchers*. Robinson and Barnett (1975) reported the case of a 17-year-old girl who had watched *Jaws* and experienced anxiety and sleep disturbances consequently. She was found the next day jerking her limbs, screaming about sharks. Turley and Derdeyn (1990) reported the case of a 13-year-old boy who became “addicted” to horror films, particularly *A Nightmare On Elm Street*. One study found that two 10-year-old boys experienced anxiety for up to 8 weeks after watching the TV program *Ghostwatch* (Simons and Silveira, 1994). Symptoms included fear of ghosts and of the dark, refusal to go upstairs alone, nightmares, sleeping with the light on, and panic attacks. Ballon and Leszcz (2007) reported the case of a 22-year-old unemployed woman with three children who were at 23 weeks’ gestation but felt possessed and had flashbacks of watching *The Exorcist*. According to the authors, all of the cases of “cinematic neurosis” they reviewed involved individuals who had experienced a recent loss (or potential loss) of a family member about whom they were ambivalent. Individuals also held strong religious or cultural ideals, and their behavior included recalling imagery from the films they had seen. The films also appeared to have some personal meaning to the individuals.

Sparks (1989a,b) found that around half of the women and the quarter of the men surveyed in his study reported enduring fright after watching horror. Women appeared to be particularly affected (Sparks et al., 1993) with around half of the women subsequently avoiding such films, 68% perceiving specific rooms as anxiety provoking (compared with 10% of men), and 43% reporting nervousness. Harrison and Cantor (1999) found that 90% of their sample of 136 young people (average age – 20.6 years) had experienced a film that was so frightening that the experience had lasted beyond the viewing of the film. More than 50% of the sample reported sleep disturbances and eating problems.

The rarity of such extreme emotion distress requiring psychiatric intervention suggests that horror film, while designed to evoke fear and panic, has no significant long-term consequences than can impair an individual’s mental, social, and occupational function and that those individuals who do report this impairment in functioning have other characteristics or have undergone other experiences, which may underlie the condition they report. While there is no evidence that exposure to horror films has adverse or sustained effects on mental health in individuals with no pre-existing mental health issue, there is evidence that watching horror films can lead to self-reported short-term anxiety and disturbed sleep.

## Development of Fear and Horror Liking/Avoidance

Children express fear to horror, just as adults do, and they also express enjoyment of horror and graphic violence, just as some adults do, and some have argued that this interest peaks at adolescence (Twitchell, 1989). The form of the stimulus children fear appears to change as they develop, with unfamiliar or threatening versions of concrete objects the source of anxiety in infancy and imaginary and symbolic stimuli the source of fear in the pre-school years. Fear stimuli become more concrete and realistic when children are at school age (Hyson, 1979). Bauer (1976) found that drawings of imaginary feared objects decreased with age (from kindergarten to age 11 or 12), whereas depictions of realistic injury increased. Fright reactions occur to violence, injury, or physical danger (Cantor and Wilson, 1988).

### Early Childhood

An early study of children’s preferences for scary movies found that 24% of 43 7–8-year olds and 13% of 46 11–12-year olds reported having nightmares, and younger girls reported more fears than did younger boys (Palmer et al., 1983). Younger boys liked scary films more than did younger girls. About 40% of the younger children liked scary programs; 65% of the older children did. Seven percent of older children and 28% of younger children disliked scary films; 68% of younger children said they avoided scary TV shows, whereas 11% of the older group did. Cantor and Reilly (1982) found that 11–12-year olds reported avoiding frightening TV and films more than did 15–16-year olds, and Cantor et al. (2010) found that the most common content causing fear in 219 8.5-year olds was the supernatural (imaginary/fictional monsters) with someone being hurt the next most common. Having a television in the bedroom was the best predictor of fright severity, and the average age of exposure to stimuli was 6.6 years; 67% were able to provide the name of the show. Seventy-one percent could not stop thinking about the experience; 52% worried about it; 36% reported shaking; 59% did not want to sleep alone; and 56% had nightmares. When another sample (*N* = 164) was asked why they watched, 40% said it was because they wanted to and 40% because someone else was watching. A study of 314 7–12-year-old Dutch children’s response to TV-induced fright found that interpersonal violence was the most fear-inducing content and fantasy the least; the films, which caused the greatest fear, had been intended for adult audiences – *Gremlins, IT, Commissaris Rex,* and *The X Files* (Valkenburg et al., 2000). Girls experienced more fear than did boys but fear in both sexes declined with age. Girls physically intervened and used social support and escape more than did boys. Cognitive reassurance was the most common coping strategy, and social support was the least common.

### Coping

How children cope with horror has been the subject of some research on child development and horror because of the potentially harmful psychological consequences of exposure to frightening stimuli. Cantor and Wilson’s (1988) review of the effect of horror stimuli on children’s behavior concluded that two methods of coping were generally employed. Non-cognitive strategies were those which did not involve the processing of verbal information and which might involve desensitization (the gradual exposure to the fear stimulus); cognitive strategies were those whereby children were encouraged to think about the source of their fear as a means of coping with the stimulus. There is evidence that desensitization is successful (Wilson and Cantor, 1987). For example, children (5–7 and 8–9 year olds) who had been gradually introduced to a videotape of snakes showed less fear when watching the snake pit scene from *Raiders Of The Lost Ark*. A similar effect was found in a study of 5–7- and 8–11-year olds in which participants played with a rubber tarantula and later saw a scene from *Kingdom Of The Spiders* (Wilson, 1987), and in a group of kindergarteners and 5–6-, 7–8-, and 6–9-year-old children who were exposed to photographs of worms and then saw a frightening film featuring worms. The children who had been previously exposed to the creatures enjoyed the film more than did those not exposed; exposure to live worms reduced the fear evoked by the film in boys (Weiss et al., 1993). Cantor et al. (1988) found that 3–5-, 6–7-, and 9–10-year-old children’s fear of the Hulk in *The Incredible Hulk* could be reduced if children saw a TV program, which showed the making of the TV series, and how the make-up of Lou Ferrigno (the actor who played the Hulk) was applied. Children of different ages become afraid at different stages of the TV program and the Hulk’s transformation (Sparks and Cantor, 1986): 3–5-year olds became more frightened after the transformation, whereas 9–11-year olds became more frightened before the transformation. Cantor et al.’s finding is also anecdotally illustrated in the preface to Englund (2009). Here, Wes Craven (the director of *A Nightmare On Elm Street*) describes filming Robert Englund (Freddy Krueger in Elm Street) explaining that he was the actor who played a character so that the video could be sent to a distressed child who found Krueger very frightening.

Younger children (4- and 5-year olds) appear to benefit from adopting more physical strategies such as holding on to a blanket/toy or eating/drinking (Wilson et al., 1987). The reasons for the success of this strategy might be the provision of relief from anxiety and the provision of tactile contact in linguistically developing children or by the occupation of working memory, which reduces the cognitive resources available to think about and process fear stimuli. Proximity to a parent is perceived as being the most successful fear-reduction coping strategy in young children (Wilson et al., 1987). Very young children (under 2 years) experience less fear through covering their eyes; in 3–5-year olds, this behavior increases fear (Wilson, 1989).

Cognitive strategies, such as talking about films and programs with parents or other adults, have been found to be effective (Cantor and Wilson, 1988). By far, the most common type of cognitive strategy employed by parents is reassuring children that the stimulus children are afraid of does not exist (Cantor and Hoffner, 1990), although this is likely to be successful in older children but not in younger children (4–5 years; Cantor and Wilson, 1984). Explaining that the source of fear is not likely to be harmful is also successful in older (8–9 year old) children (Wilson and Cantor, 1987). Wilson and Cantor’s study, which involved informing children that most snakes were not poisonous and telling them about the behavior of snakes, found that these instructions increased fear in 5–7-year olds. Verbal explanations may be ineffective in younger children who are less likely to discuss horror materials with their parents. Cantor et al. (1986) found that none of their 3–7-year-old children discussed a film with parents, but 43% of 8–12-year olds and 50% of 13–18-year olds did. However, verbal priming prior to seeing the film can sometimes increase the child’s emotional response to what they see (Cantor et al., 1984). If children are informed that a film has a happy ending, they report less fear (Hoffner and Cantor, 1990; Hoffner, 1997). Introducing probability information about events prior to watching a film such as telling children the likelihood of an event occurring appears to have no effect on 5–9-year olds’ emotional response (Cantor and Hoffner, 1990). If children rehearse verbal information (e.g., “this tarantula cannot hurt people; they are not poisonous”), older and younger children respond less emotionally to a film about tarantulas (Wilson, 1987). Children also regard the spiders as less dangerous after being given these instructions.

Two physical means of coping with frightening stimuli studied in children are blunting (avoiding threat or transforming a threat by distraction; looking away, for example) and monitoring (being action oriented and attending to the threat). Sparks and Spirek (1988) found that high blunters and low monitors were less physiologically aroused by horror films than were high monitors and low blunters suggesting that underlying physiology might predict or predispose individuals to react in a given emotional way to frightening stimuli; Sparks (1989a,b) also found that low monitors were less negative about horror when given information about the film but this information had no effect on blunters. A study of 228 14–15- and 15–16-year olds examined the role of blunting and monitoring on coping with scary films (Hoffner, 1995). Hoffner investigated empathetic concern (EC, other-oriented) and personal distress (PD, feelings of anxiety/discomfort in response to suffering) by examining four coping methods – interpersonal comfort (IC), distraction (D), momentary avoidance (MA), and unreality. Davis and Kraus (1997) had previously reported that high empathetic concern was associated with less loneliness and unsociability; high personal distress was associated with shyness, poor interpersonal functioning, and social anxiety. Empathetic concern was found to encourage altruism, whereas personal distress prompted people to reduce their own emotion expression (Batson, 1987).

Hoffner found a series of interesting results. A belief that something was unreal was the most common coping strategy, followed by interpersonal comfort and momentary avoidance; these were used more than was distraction. About 50% of the sample considered unreality and momentary avoidance to be effective; 26% considered distraction to be effective. The study found that boys preferred scary films more than did girls, a finding consistent with the literature, that girls reported more empathetic concern and personal distress, that personal distress correlated with empathy and with monitoring and blunting, that these correlated negatively with liking for scary films, that blunting predicted use of distraction and unreality, that monitoring was more widely used and was more effective, that monitoring and blunting were associated with increased interpersonal comfort, that girls were more likely to use momentary avoidance and interpersonal comfort and consider them more effective, that people who reported using one strategy were more likely to use all four, that empathy, but not personal distress, was associated with greater use of reality, IC and personal distress were associated with increased use of distraction, and that higher empathy scores were associated with greater use of Unreality. People who liked horror were less likely to use distraction, unreality, and momentary avoidance as coping strategies, which suggest that coping is related to the dislike of horror – it is something that must be done to mitigate the effects of something that is disliked. If people thought the coping strategies worked, they enjoyed the films more.

Hoffner also noted that participants who reported finding scary films and television to be violent were likely to use all four coping mechanisms; those who found the material to be realistic were more likely to report using distraction, unreality, and interpersonal comfort as coping mechanisms. Material featuring blood and gore was more likely to lead to the use of momentary avoidance. Girls reported using momentary avoidance and interpersonal comfort more than did boys and considered these to be more effective strategies than did boys.

### Adolescence

As children enter adolescence, their reasons for seeking out horror develop and change – they will watch to be thrilled, to rebel (because parents have prohibited them), or to enjoy gore because they are interested in how people die (Oliver, 1993a,b). One study of 220 13–16-year-old boys and girls examined their motivation for watching slasher movies (Johnston, 1995). Reasons for watching included gore watching, thrill watching, an increased feeling of independence bravery, and problem avoidance. Thrill watching and independence were positively related to positive affect; positive views of slashers were associated with high gore and thrill watching and gore watching predicted preference for graphic violence. Boys were more likely to watch graphic horror because they were motivated to seek out gore, and they were also more likely to identify with the killer than were girls; girls were more likely to identify with the victim. A larger survey of 6,522 10–14-year-old US adolescents in 2003 found similar sex differences: watching violent films was associated with being male, older, non-white, having less educated parents, and having poor school achievement (Worth et al., 2008); teenage boys in another study who were regarded as aggressive and excitable found violent cartoons to be as funny or thrilling (Aluja-Fabregat and Torrubia-Beltri, 1998). Both boys and girls who found violent cartoons funny and thrilling also scored higher on neuroticism, psychoticism, and sensation seeking.

### Aging and Horror Enjoyment

The majority of the research on the development of horror preference and response to horror film has recruited children and adolescents as participants. There is very little research on how horror film and horror media in general are perceived as individual’s age and approach caducity, a paucity that is also reflected in humor research. There is some, but not much, research on how older people respond to horror, and this suggests that the preference for horror declines with age (Tamborini and Stiff, 1987; Hoffner and Levine, 2005). Clasen et al. (2019), for example, found a negative correlation between age and enjoyment of horror media and horror use suggesting that both decline as we age. As Clasen et al. concede, however, their sample was clustered around the 35-year age. The average age of those who agreed that they strongly liked horror media was slightly lower than those who disagreed (33.5 vs. 36.5 years). They also note that since sensation seeking also declines with age, this might explain the reduction in enjoyment and seeking out of horror with increasing age post adolescence.

The literature from developmental research mirrors the findings from that in the adult sex differences research in that boys prefer, and seek out, horrifying/scary material more than do girls. Children tend to express greater fear to different types of stimuli and content depending on the age of the child. There are also differences between boys and girls (and between age groups) in the types of coping strategies they adopt during and after watching frightening television and film material. Cognitive strategies, in particular, have been found to be effective with talking about film content and explaining that “monsters” do not exist or that the characters can actually cause no harm being the most effective.

## What Causes Fear?

One of the principal purposes of horror film is to induce fear. The nature of fear and its etiology has a long history in psychology, and various models have been proposed, which have attempted to explain why we become afraid and to what types of stimulus. One model, for example, has proposed that we have evolved a “fear module,” a theoretical construct, which comprises a number of domain-specific programs and which is “preferentially activated … by stimuli that are fear relevant in an evolutionary perspective” (Öhman and Mineka, 2001). Fear, it is argued, motivates us to escape and escape very quickly from potential threat and threats to survival (Mineka and Öhman, 2002). The module has four features: it is selective, it is automatic (when encountering fear-relevant stimuli, it responds without mediation), it is encapsulated (i.e., it relies on proven strategies to deal with threat), and it is underpinned by specific neural behavior (Öhman and Mineka, 2001). It is considered to be an adaptive mechanism for allowing us to avoid physical danger rapidly (Schaller and Neuberg, 2012). In the context of horror film, this is, of course, counter-intuitive as horror film viewers who enjoy horror may not wish to escape the horror and deliberately and proactively approach and seek it, and those that do not enjoy horror and who may serendipitously watch horror engage in other withdrawal behaviors such as shutting the eyes or holding on to a companion (they may also leave a cinema or turn off a screen). What occurs during horror film viewing is the willing acceptance that the film will induce fear and that a contract is reached between the medium’s manufacturer and the viewer that this is what is to be expected. The questions that then arise are whether there are specific stimuli or situations, which horror films deploy or recruit which are more likely to induce a fear response and, if so, what are these stimuli and why do they have this effect.

Mineka and Ohman’s conceptualization draws on the (controversial) notion that there are some stimuli to which we are evolutionarily predisposed to fear – that evolution has rendered us more afraid of some objects and situations – and there are stimuli to which we have become socially or cognitively conditioned to fear (e.g., examinations, being in objectively non-threatening social groups). The latter stimuli pose no immediate and real physical threat to survival (i.e., they are not fatal), but the former may potentially present this threat by endangering or causing death, may generate threat, and, therefore, make us more alert to our environment, and these stimuli and situations were experienced by “pre-technological” humans (Seligman, 1971). These stimuli and situations were those which once posed threats to our ancestors and that we, therefore, developed an evolutionary disposition to avoid or to respond with fear, a form of selective association. Guns, for example, are not fatal unless used, and our exposure to them is limited; guns are not phobic stimuli and seeing photographs of guns – or seeing guns – does not elicit significant fear, and not the degree of fear that stimuli to which we are evolutionarily predisposed to fear evoke. A person pointing a gun at us, however, with the intention to fire or with the threat of the intention to fire is clearly a direct threat but not one that is evolutionarily created.

One of most common phobias is arachnophobia, and spiders have been a staple of horror films since the 1950s, although only 0.1–0.3% of spider species are venomous (Gerdes et al., 2009) and conditioned fear to spiders is very difficult to extinguish (Davey, 1994). Individuals are faster at detecting images of spiders and snakes among innocuous stimuli than they are innocuous stimuli placed in an array of threatening stimuli (Öhman et al., 2001). This predisposition facilitates vigilance (occasionally, over-vigilance and we see threat in ambiguous situations) to sources of threat or danger with greater attention paid to some stimuli (Clasen, 2014; March et al., 2017). It is a self-protection and survival-enabling mechanism motivating us to confront (and, therefore, remove the potential source of threat) or flee (thereby, removing us from the context in which a threat could result in endangerment).

Fear is related to expressions of disgust, and the literature on phobia suggests that the strength of fear for phobic objects is closely related to disgust sensitivity but not trait anxiety (Davey, 1994) such that people who express abnormal fear of an object also show high degrees of sensitivity to disgusting stimuli but are not dispositionally, highly anxious. A specific phobia, which appears to be qualitatively and quantitatively different from others and is relevant in the context of horror film, is the fear of blood or blood-injection-injury phobia (Wani et al., 2014; Brinkmann et al., 2017). This accounts for 3–4% of phobias and is characterized by fear of blood withdrawal, medical intervention, and seeing others’ blood (Brinkmann et al., 2017). Vasovagal syncope (fainting due to low blood pressure and heart rate caused by exposure to a stimulus) is seen in 75% of phobic individuals – there is a short increase followed by a decrease in heart rate. Individuals experience fear, anxiety, and disgust and avoid or decline medical treatment because of the strength of their phobic reaction (Wani et al., 2014). This extreme experience may explain why some people feel squeamish at the sight of blood in horror: blood is unique as a stimulus, which evokes a strong fear or disgust reaction.

## Neuropsychology and Horror Film

Fear is the most widely studied emotion in science because it can be easily conditioned, studied, and observed in non-human organisms. There is a substantial literature, which has attempted to explain fear conditioning and learning through reference to its underlying neuropsychology, and much of this work has been conducted on non-human species (LeDoux and Hofmann, 2018). In humans, much of our understanding of the neurology of fear has derived from neuroimaging research and studies of brain injury. One of the brain regions involved in fear recognition and experience is the amygdala (Martin, 2008; March et al., 2017), and a considerable literature exists examining the role of this structure in the conditioning and maintenance of fear.

No study has specifically examined the effect of exposure to horror film on brain activation, although hundreds of studies have examined the effect of exposure of fear-related stimuli, including films designed to induce fear, on brain activation measured *via* MEG, PET, fMRI, and EEG. Many studies have examined the consequence of brain injury on the fear response, and one study is especially relevant to horror film as it examined the effect of bilateral amygdala injury on responses to fear-related stimuli in a film-related context (Feinstein et al., 2011).

In this study, a 44-year-old woman with normal IQ and language showed impaired fear conditioning, impaired recognition of fear in faces, and impaired social-related fear. Feinstein et al. attempted to induce fear by taking her to the pet shop where there were snakes and spiders, walking her through a haunted house, and having her watch horror films. Although she verbally indicated avoidance of the spiders she physically approached them and asked 15 times if she could touch one; at the haunted house (a visitor attraction), she volunteered to lead a group of visitors, did not hesitate in walking around, and was not scared by the monsters (she scared the actors). None of the 10 horror film clips elicited fear (other film clips designed to elicit other emotions successfully elicited those emotions) and she asked for the name of one so that she could rent it. She recognized that most people would be scared by them. This is only comprehensive study of the effect of region-relevant brain injury on the perception of horror films and horror-related stimuli in a single-case study, and while single case studies need to be interpreted cautiously, the study does provide the opening for other studies to confirm the role of these structures in horror appreciation. One possible extension of this study would be to examine whether amygdala reactivity is associated with enjoyment of horror film (those with highly reactive amygdalae may fear or enjoy horror more than those with less reactive amygdalae) or whether the amygdala becomes increasingly active with greater stimulation, and the intensity of the experience correlates with the increase in activity while watching.

## Conclusions

The current review sought to determine why people watch horror film and how exposure to horror film affects behavior. Based on the literature from various disciplines, the following conclusions can be reached: (1) low empathy and fearfulness are associated with more enjoyment and desire to watch horror; (2) specific dimensions of empathy are better predictors of people’s responses than are others, but these dimensions are inconsistently predictive; (3) empathetic concern and personal distress are negatively correlated with enjoyment of horror involving torture; (4) there is a positive relationship between sensation seeking and horror enjoyment/preference, but this relationship is not consistent and may depend on the component of sensation seeking; (5) men and boys prefer to watch – and enjoy and seek out – horror more than do women and girls; (6) women and girls report experiencing more fear and anxiety generally than do men and express greater anxiety and fear when watching horror than do boys and men; (7) this sex difference may be attributable to women’s typical higher disgust sensitivity and anxiety proneness (both of which are inter-related); (8) women report more empathetic concern than do men, and this may be another explanatory mechanism; (9) no study to date has systematically explored disgust sensitivity as a mediator in horror enjoyment and preference, but the evidence would suggest that the former will predict the latter; (10) older children are more afraid of concrete objects/stimuli when very young but of symbolic stimuli when younger; (11) individuals tend to prefer horror less as they age, but there is little literature on this topic; (12) children use various coping strategies to overcome horror film-related fear and the success of these depends on the age of the child; (13) physical coping strategies are more successful in younger children; (14) priming with information about the feared object helps reduce fear and increase enjoyment when children watch a film featuring the feared stimulus; (15) the startle reflex is amplified in the presence of threatening stimuli; and (16) little is understood about the role of neuropsychology in the response to horror film generally although the understanding of the structures and regions of the brain implicated in fear and fear conditioning is well documented; the amygdala is likely to be involved in the reaction to (and enjoyment of) horror.

## Limitations and Future Directions

The conclusions in the previous paragraph are based on a very limited set of data. The studies from which such data have been drawn have varied in sample size, methodology, and materials, and these are three clearly identifiable and major limitations in this field. Hoffner and Levine (2005) have highlighted similar limitations in their meta-analysis. The type and selection of stimuli used in behavioral studies of horror film and researchers’ definition of what constitutes a “horror” or “graphic” horror film has led to a literature, which renders making generalizations about horror’s effects difficult, the summary above notwithstanding. Studies have used a variety – although a very restricted variety – of horror films over 30 years of research, and the films share little in common apart from being classed as horror film. *The Silence of the Lambs, Cannibal Holocaust, The Babadook, Saw, The Blair Witch Project, Psycho, Dracula*, and *The Devil Rides Out* are all horror films, but each has distinctive mechanisms of evoking fear and disgust based on story, film making, plot, characters, sound, performance, visual effects, credibility, and use of music. No one study can fully take into account our response to horror because not all horror films are the same (Oliver, 1993a,b), and this limitation needs to be more clearly recognized and addressed in future work.

Hoffner and Levine (2005) have concluded that the nature of the media content in these studies can explain the failure to find homogeneity in the correlations between enjoyment of horror media and empathic concern in their meta-analysis. As noted earlier, when correlations were found for empathy and horror enjoyment, the most consistent correlations found were in those studies in which victimization formed the dominant aspect of the horror stimuli. When these studies were removed, the correlations for the remaining studies fell to almost zero. These studies measured participants’ responses to the enjoyment of horror film as a genre (or response to a drama with a likeable victim), rather than their responses to specific horror films or their experience of watching specific horror films. Hoffner and Levine’s analysis identifies at least two limitations in the field noted here: the heterogeneity of the material used as stimuli in experiments, and the nature of the question asked in these studies (for example, whether the question is: do you enjoy this specific film/film clip? or Do you enjoy this genre of film?). The former limitation can be easily resolved *via* empirical research. Studies, for example, might examine the role of the nature of the character, the narrative drive of a film (point of view), the esthetics of the film, a film’s use of music, the number acts of violence, and the types of acts of graphic violence and the perpetrator of the violence, the characteristics of the perpetrator, and the victim (their attractiveness, age and sex, for example), a film’s use of color and the use of specific tropes and techniques (such as found-footage and types of horror film). This is not to say that some of these elements have not been studied – this review and others have described studies in which they have – but there has been little research which has examined these elements systematically and methodically, and some elements have not been explored at all.

The issue of self-report – and self-report based on very small samples – is another possible limitation in that authors rely on individuals’ subjective reports based on their impressions and perceptions, and these reports are based on responses to standard questionnaires or questionnaires developed by the authors. This is an issue for any research, which aims to determine how people think and feel and is currently the most effective way of measuring people’s responses. It is possible to study non-verbal measures (such as movement, EEG, brain activation, GSR, and so on), but these are indirect, correlational measures of what an individual might be thinking or feeling. Motor behavior, however, may be a very informative indicator of response to horror, as some of the studies reviewed here suggest.

Given the current accessibility of film and media generally *via* smartphones, as well as internet-ready TVs and, of course, computers, one topic of research that has been little studied is whether the medium affects the perception and enjoyment of horror films. Filmmakers may bemoan the viewing of material on a smartphone that was designed for a screen that is 1,000 times larger, but it would be instructive to examine whether screen size affects people’s esthetic, emotional, and cognitive response to horror. Screen size and its effect on the enjoyment of displayed material have been relatively well-studied (see, for example, Grabe et al., 1999; Lombard et al., 2000; Rigby et al., 2016). In the context of horror, however, it is hypothesizable that increased screen size leads to increased visibility and that this would result in a stronger fright reaction because more of the horror can be seen and seen more clearly. It is also possible that the augmentation of the screen would also augment the sound (an auditory-sound illusion) so that bigger screens might affect our perception of horror because of this visual illusion.

There is also scope for further research on coping with the effects of watching horror film and of mitigating the fright if the experience is considered too intense or too unmanageable. Of course, individuals could choose not to watch or could chose to watch selectively if they are in front of the screen. But there may be more imaginative strategies that might be adopted such as the introduction of non-visual, non-verbal, and non-auditory stimuli (e.g., scent). It is possible that the presence of a pleasant scent might alleviate some of the fright generated by horror film if such alleviation is required (either because it distracts or because it creates or elevates positive mood). There is some evidence that this might be possible (Martin, 2013), and this is a question that merits pursuit. Wes Craven’s film, *The Last House On The Left*, utilized a similar, if non-olfactory distraction technique in the tagline for the film, which was “Keep repeating, it’s only a film…it’s only a film…”

The majority of the studies reviewed here has included mono-cultural samples, and the current review was unable to uncover any cross-cultural research on horror enjoyment or preference. An understanding of the cultural influences on film preference (especially horror) and the individual differences that may underpin them warrants investigation given that certain genres of horror appear to be more popular and appear more often, in specific cultures: Different cultures place different emphases on certain types of content and Japanese horror with its emphasis on ghosts, the supernatural is an obvious example (Balmain, 2008; McRoy, 2008). Others have argued that the European horror film is distinct from other types of horror film and has a specific “esthetic” (Allmer et al., 2012). There is a considerable literature on the difference between collectivistic and individualistic cultures with research suggesting that the psychological responses of individuals from each type of cultural background are different (Matsumoto et al., 2008; Alotaibi et al., 2017; Gendron, 2017). In the field of horror film perception, experience, and enjoyment, it could be hypothesized that individuals from collectivistic cultures might respond differently to horror (and victims in horror) than do individuals from individualistic cultures – specifically individuals from collectivistic cultures may express greater fear compared to those from individualistic cultures – and this is an hypothesis that can be easily tested.

With interest and appreciation in horror increasing, the scope for undertaking research into horror film has never been more timely. There is still much to discover and still much to understand. Horror, said Adorno in another context, was beyond the scope of psychology. The research would suggest that the weight of evidence is on the side of one of horror’s innovators. Without psychology, Dario Argento once said, the horror film does not exist.

## Author Contributions

The author confirms being the sole contributor of this work and has approved it for publication.

### Conflict of Interest

The author declares that the research was conducted in the absence of any commercial or financial relationships that could be construed as a potential conflict of interest.
